# Population genomic footprints of selection and associations with climate in natural populations of *Arabidopsis halleri* from the Alps

**DOI:** 10.1111/mec.12521

**Published:** 2013-10-28

**Authors:** Martin C Fischer, Christian Rellstab, Andrew Tedder, Stefan Zoller, Felix Gugerli, Kentaro K Shimizu, Rolf Holderegger, Alex Widmer

**Affiliations:** * ETH Zürich, Institute of Integrative BiologyUniversitätstrasse 16, 8092, Zürich, Switzerland; † WSL Swiss Federal Research InstituteZürcherstrasse 111, 8903, Birmensdorf, Switzerland; ‡ Institute of Evolutionary Biology and Environmental Studies and Institute of Plant Biology, University of ZurichWinterthurerstrasse 190, 8057 Zürich, Switzerland; § ETH Zürich, Genetic Diversity CentreUniversitätstrasse 16, 8092, Zürich, Switzerland

**Keywords:** adaptation, environmental association, genome resequencing, pooled sequencing, Pool-Seq, population genomics

## Abstract

Natural genetic variation is essential for the adaptation of organisms to their local environment and to changing environmental conditions. Here, we examine genomewide patterns of nucleotide variation in natural populations of the outcrossing herb *Arabidopsis halleri* and associations with climatic variation among populations in the Alps. Using a pooled population sequencing (Pool-Seq) approach, we discovered more than two million SNPs in five natural populations and identified highly differentiated genomic regions and SNPs using *F*_ST_-based analyses. We tested only the most strongly differentiated SNPs for associations with a nonredundant set of environmental factors using partial Mantel tests to identify topo-climatic factors that may underlie the observed footprints of selection. Possible functions of genes showing signatures of selection were identified by Gene Ontology analysis. We found 175 genes to be highly associated with one or more of the five tested topo-climatic factors. Of these, 23.4% had unknown functions. Genetic variation in four candidate genes was strongly associated with site water balance and solar radiation, and functional annotations were congruent with these environmental factors. Our results provide a genomewide perspective on the distribution of adaptive genetic variation in natural plant populations from a highly diverse and heterogeneous alpine environment.

## Introduction

Studies of genes and genomic regions underlying adaptation are at the heart of ecological genomics (Bergelson & Roux 2010; Ekblom & Galindo 2011), a dynamic research area that aims at understanding the genomic changes associated with the responses of organisms to their biotic and abiotic environment (Ungerer *et al*. 2008). Natural genetic variation is essential for adaptation to heterogeneous environments and to changing climatic conditions (Hoffmann & Sgro 2011), and adaptive divergence can initiate the speciation process by forming genomic islands of divergence (Feder *et al*. 2012). Little is known about the effects of natural selection on the amount and distribution of adaptive genetic variation, that is, genetic variation associated with fitness, within and among natural populations of most organisms (Holderegger *et al*. 2006). Numerous methods have been developed for detecting the signature of natural selection (see e.g. Oleksyk *et al*. 2010), and the field of ecological genetics is shifting from the analysis of a limited number of anonymous markers or candidate genes to truly genomewide studies that encompass thousands of single nucleotide polymorphisms (SNPs) or entire genomes (Andrew *et al*. 2013). Such genomic analyses are aided by progress in next-generation sequencing (NGS) technology, which allows for the expansion of genomic analyses to organisms for which no reference genome is available (Davey *et al*. 2011; Everett *et al*. 2011).

A major difficulty associated with this technology is that population genetic approaches typically require sampling of multiple individuals from several populations. Recently, it has been shown that NGS analysis of pools of individuals (Pool-Seq) is effective for SNP discovery and provides accurate estimates of allele frequencies (see e.g. Futschik & Schlötterer 2010; Gautier *et al*. 2013; Rellstab *et al*. in press). The pooling of multiple individuals per population provides a cost-effective approach to estimate genomic diversity at the population level, and it should thus also be suitable for the identification of candidate genes through approaches such as genomewide environmental associations (Schoville *et al*. 2012).

*Arabidopsis halleri* (L.) O'Kane & Al-Shehbaz (Al-Shehbaz & O'Kane 2002) is ideally suited for population genomic studies of nucleotide variation in natural populations, because it is an emerging model plant species in ecology and evolution (e.g. Meyer *et al*. 2009; Hanikenne & Nouet 2011) and it is closely related to the plant model *A. thaliana* (Clauss & Koch 2006). In addition, its genome of 255 Mbp is relatively small (Johnston *et al*. 2005). *Arabidopsis halleri* grows in a wide range of habitats, typically at 600 m above sea level (asl) and higher, including mountain slopes at more than 2300 m asl, grassy meadows, forest margins and rocky crevices (Al-Shehbaz & O'Kane 2002) on soils with acidic, neutral and oligotrophic properties, and on soils with high natural or anthropogenic heavy metal content (Clauss & Koch 2006). It is a perennial, outcrossing and insect-pollinated herb that is distributed throughout Europe and eastern Asia. Recent studies have investigated a wide range of ecological and evolutionary questions, including population history (e.g. Heidel *et al*. 2010; Pauwels *et al*. 2012), self-incompatibility (e.g. Roux *et al*. 2013), speciation (e.g. Shimizu-Inatsugi *et al*. 2009; Roux *et al*. 2011) and heavy metal tolerance (e.g. Hanikenne *et al*. 2008; Meyer *et al*. 2009). The ability of *A. halleri* to grow in a wide diversity of habitats and our increasing understanding of its biology make it an ideal study object for analyses of adaptation to different edaphic and climatic conditions.

Plant populations growing in the Alps are exposed to a wide range of often stressful abiotic and biotic environmental conditions that may change dramatically with altitude and aspect (Körner 2007). Such steep ecological and environmental gradients may cause strong selection and lead to adaptation over small geographical distance. At this scale, gene flow may be more effective at countering selection than at larger scales, which may lead to distinct footprints of selection across the genome. Thus, the study of *A. halleri* populations from contrasting habitats in the Alps offers excellent opportunities for detecting genes and genomic regions affected by environment-mediated selection.

In the present study, we investigated geographically close natural populations of *A. halleri* growing in heterogeneous alpine environments from a population genomic and ecological perspective. Our goals were to characterize genetic variation and population genomic footprints of selection across the genome of an alpine plant species to identify genes that reveal associations with climatic variation between our study populations. Specifically, we asked 1) what proportion of SNPs show highly elevated differentiation across the studied populations, 2) what functional categories are overrepresented among the genes containing highly differentiated SNPs, 3) what proportion of these genes are associated with the studied abiotic topo-climatic factors, and 4) whether such genes also have functions consistent with their associations with environmental factors.

## Materials and methods

### Selection of populations and environmental factors

**Figure 1:**
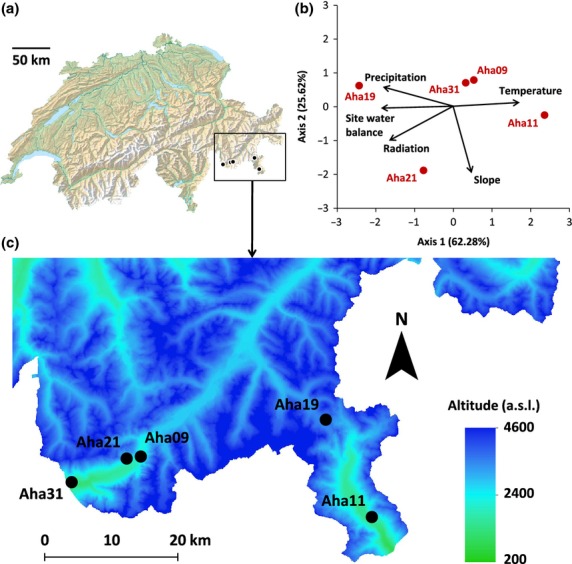
(a) Location of the five sequenced populations of *Arabidopsis halleri* in Switzerland. (b) Principle component analysis of the five populations using five environmental factors (Table S1, Supporting information). Environmental factor coordinates (arrows) were multiplied by two for clarity. (c) The locations of the studied populations (black dots) in the south-eastern Swiss Alps (Digital Elevation Model DHM25 L2, reproduced by permission of swisstopo [JA100118]).

Populations of *A. halleri* were collected from five locations in the south-eastern Swiss Alps representing a wide variation in environmental factors (Fig.1, Table1). Populations were situated in close geographical proximity to minimize potential confounding effects of population structure owing to neutral demographic processes and population history. To characterize the habitats at the sampled *A. halleri* populations with respect to abiotic environmental factors, interpolated GIS data (ARCMAP 10; ESRI) were extracted for 21 topo-climatic factors collected over a 30-year period (1961–1990) at 25-m resolution (Zimmermann & Kienast 1999). Average values for each climatic factor were used for analysis. We first conducted pairwise correlation analyses (Pearson's *r*) with IBM SPSS Statistics 19 to remove highly correlated factors (|*r*| ≥ 0.8). This led to the selection of five topo-climatic factors, describing precipitation (PRECYY), slope (SLP25), solar radiation (SRADYY), site water balance (SWB) and temperature (TAVEYY; for details see Tables1 and S1, Supporting information). To illustrate environmental variation between the sampling locations, we performed a principle component analysis (PCA) with STATISTICA 10 (StatSoft), using a generalized inverse correlation matrix (Fig.1b).

**Table 1:** Sampling locations of *Arabidopsis halleri* populations and their topo-climatic characterization. For details on topo-climatic factors, see Table S1 (Supporting information)

Population	Location	Coordinates (°N/°E)	Altitude (m asl)	PRECYY (Precipitation) (1/10 mm/year)	SLP25 (Slope) (°)	SRADYY (Radiation) (kJ/m^2^/day)	SWB (Site water balance) (1/10 mm/year)	TAVEYY (Temperature) (1/100 °C)
Aha09	Vicosoprano-1	46.36925/9.65868	1403	11 368	6	16 865	−44	527
Aha11	Brusio	46.27767/10.10619	1070	10 561	15	16 654	−1118	810
Aha19	Poschiavo	46.41125/10.02253	2308	15 564	5	19 180	380	60
Aha21	Vicosoprano-2	46.36682/9.63081	1610	12 202	21	19 780	231	431
Aha31	Castasegna	46.33682/9.52171	790	13 133	6	18 000	−163	892

### DNA extraction and genome resequencing

Tissue from 20 individuals per population was collected at distances >4 m in summer 2010 and 2011. Genomic DNA of each individual was extracted from dried leaves using the DNeasy Plant Kit (Qiagen). DNA quality was checked on 1.5% agarose gels stained with GelRed (Biotium) on a UV-Vis Spectrometer (NanoDrop 8000, Thermo Scientific). DNA quantity was measured with a Qubit fluorometer (dsDNA BR, Invitrogen). Equal amounts of high-quality DNA from 20 individuals per population were then pooled, resulting in a pool size of 7 μg RNA-free genomic DNA per population. Library preparation (250–300 bp insertion size; 100 bp paired-end reads) and sequencing on an Illumina HiSeq2000 were performed by GATC Biotech (Constance, Germany).

### Illumina read processing

Forward and reverse reads were screened for tags and adaptors and trimmed with CUTADAPT (Martin 2011). Phred-type quality scores Q20 were used for quality trimming with the FASTX toolkit (http://hannonlab.cshl.edu/fastx_toolkit). Files containing forward and reverse reads were resynchronized with an in-house perl script, and only paired sequences were used for further analysis.

### Read mapping and SNP calling

Trimmed paired-end reads of each population were mapped to the *A. thaliana* reference genome (TAIR10, Kaul *et al*. 2000; Lamesch *et al*. 2012), and organellar DNA was excluded. Reads were mapped using BWA aln and sampe (Li & Durbin 2009), allowing 10% mismatch with the *A. thaliana* reference genome. PCR duplicates (2.03–4.34%) and ambiguously mapped reads (2.54–2.97%) were rare. The latter were removed before the remaining high-quality reads were sorted with SAMTOOLS v0.1.18 (Li *et al*. 2009).

Single nucleotide polymorphisms were called with SAMTOOLS (mpileup, Li *et al*. 2009), synchronizing and filtering for base quality (Q20) with the perl script mpileup2sync.pl of POPOOLATION2 (Kofler *et al*. 2011). The minimum count of the minor allele was set to four to account for sequencing errors, and minimum coverage of 20 and maximum coverage of 400 per population were used as thresholds for SNP identification to accurately estimate allele frequencies and correct for potential errors from repeated sequences. SNP allele frequencies were estimated with snp-frequency-diff.pl in POPOOLATION2.

Highly differentiated SNPs were identified in a three-step procedure. First, genetic differentiation (*F*_ST_) was calculated with fst-sliding.pl in POPOOLATION2 according to Hartl and Clark (2007) using a sliding-window approach with a window size of 500 bp and step size 250 bp to identify SNPs and genomic regions with elevated genetic differentiation between populations. Within each sliding window, at least 50% of SNPs had to fulfil the coverage threshold specified above. Pool size per population was set to 40 because 20 diploid genomes were represented in each pool. Using this *F*_ST_ sliding-window approach, we identified the 0.1% genomic regions with highest differentiation in at least one of the ten pairwise comparisons, corresponding to a sliding-window *F*_ST_ threshold of 0.473. Such peaks with highly elevated differentiation can be a consequence of divergent selection or genetic hitchhiking (Strasburg *et al*. 2012). Second, pairwise *F*_ST_ values were calculated for all SNPs to identify those with elevated differentiation between populations. We selected the 0.1% most strongly differentiated SNPs occurring at least in one pairwise population comparison, corresponding to an *F*_ST_ threshold of 0.545. Only SNPs that fulfilled both criteria, that is, were in the 0.1% most strongly differentiated sliding windows and among the 0.1% SNPs, were further considered. To correct for neutral genetic structure, we adjusted the *F*_ST_ threshold (0.545) of single SNPs for each pairwise population comparison by adding or subtracting the deviation of the pairwise comparison from the mean neutral population differentiation based on all >2 million SNPs (see Results and Table S2, Supporting information). The corrected threshold range of specific *F*_ST_ for pairwise comparisons fell between 0.539 and 0.561 (Table S2, Supporting information). As overall average pairwise population differentiation was low (mean *F*_ST _= 0.038, range: 0.026–0.048), this provided only a weak correction but may reduce the rate of false positives as a consequence of population structure (Excoffier *et al*. 2009). Third, strongly differentiated SNPs were inspected with Fisher's exact test (Fisher 1922), implemented in fisher-test.pl of POPOOLATION2, to identify significant differences in allele frequencies between population pairs that were not biased by low coverage (Kofler *et al*. 2011). As a threshold, we used the Bonferroni-corrected *P*-value for multiple testing at α = 5% i.e. 2.39*10^−9^. We thus set highly stringent thresholds throughout (i.e. 99.9% quantiles and Bonferroni-corrected *P*-values) for identifying highly differentiated SNPs to reduce the rate of false positives.

To assess the number of genes covered by our Pool-Seq approach, we used the mpileup file produced for SNP identification, filtered for all positions that had a minimum coverage of 20x for each of the five populations and annotated them using an in-house perl script with the TAIR10 (The Arabidopsis Information Resource; http://www.arabidopsis.org) and NCBI (http://www.ncbi.nlm.nih.gov) reference databases containing 33 323 genes (including transposable elements, pseudogenes and ncRNAs). We identified 25 764 genes in our *A. halleri* data set that fulfilled these criteria for at least part of each gene.

### Environmental association analyses

We performed partial Mantel tests to check for associations between our highly differentiated SNPs and the five environmental factors, while controlling for population structure. This method has been successfully used in previous environmental association studies (Hancock *et al*. 2011; Nosil *et al*. 2012) and allows two pairwise distance matrices to be compared, while controlling for the effect of a third. In our case, the dependent variable in the model was the pairwise population *F*_ST_ matrix at a given outlier SNP, the predictor variable was the corresponding pairwise distance matrix of the environmental factor, and the third matrix contained genomewide average pairwise *F*_ST_ values to account for neutral population structure. Partial Mantel tests were run using Pearson's *r*, thereby assuming a linear relationship between allele frequencies and environmental factors. Linear methods, including linear regressions (e.g. Manel *et al*. 2010; Poncet *et al*. 2010) or variants thereof, such as linear mixed models (e.g. Coop *et al*. 2010), are widely used for association studies in ecological genomics (Schoville *et al*. 2012). Schmidt *et al*. (2008) provided theoretical and empirical support for a linear relationship between environmental factors along ecological gradients and allele frequencies. Moreover, in the analysis of a classical text book example of plant adaptation, Kooyers and Olsen (2012) found strong linear association of allele frequencies at cyanogenesis genes with minimum winter temperature in *Trifolium repens*.

Partial Mantel tests were performed with Ecodist 1.2.7 (Goslee & Urban 2007) in R v2.14 (R Development Core Team 2011; subsequent analyses were performed in R) with 1001 permutations. We used effect size (*r*) as a threshold criterion. We first performed partial Mantel tests between 20 000 matrices with simulated pairwise *F*_ST_ values (random values between 0 and 1) and the five environmental factors (real values), again controlling for population structure using (real) genomewide pairwise *F*_ST_. Then, we used the 99% quantile of the resulting 100 000 *r* values (i.e. 0.7573) as our threshold for *r*. Above this threshold, a specific SNP locus was considered to be associated with the respective environmental factor. In addition to counting the number of associations between environmental factors and individual SNPs, we assessed the numbers of associations with particular genes, that is, those that could be annotated to the *A. thaliana* genome (see above) and for which we found at least one SNP being associated with a particular environmental factor.

### Gene Ontology and functional annotation

The top candidate genes for associations with climate were selected from Gene Ontology (GO)-categories with biological functions relating to our five abiotic environmental factors, including response to temperature stimulus (GO:0009266), water stimulus (GO:0009415), osmotic stress (GO:0006970) and radiation (GO:0009314). Genes without significant association with at least one environmental factor were removed. This stringent approach did not identify genes associated with biotic factors or some of the excluded topo-climatic factors, as well as genes with unknown functions, but allowed us instead to identify candidate genes whose functions correspond to the environmental factors they are associated with.

### Molecular variation in candidate genes

We assessed whether SNP variation in our candidate genes was synonymous or nonsynonymous. As individual haplotypes are unknown in a Pool-Seq approach, we defined major allele consensus haplotypes (MACHs). These represent the consensus multilocus genotype for each candidate gene and population and were created by an in-house perl script, taking the nucleotide at each position with the highest frequency within a population pool (i.e. >50%). MACHs were aligned to their *A. thaliana* counterparts, and noncoding regions were removed using an in-house workflow and the MAFFT sequence alignment program (Katoh *et al*. 2002). Each gene was checked for premature stop codons and for sequence errors. Highly differentiated SNPs in candidate genes were then classified as either synonymous or nonsynonymous.

### Gene Ontology enrichment analysis

To explore in which biological processes genes containing highly differentiated SNPs are involved, we performed a GO enrichment analysis using topGO (Alexa *et al*. 2006). Genes were annotated with locus identifier information from TAIR, and the 25 764 *A. halleri* genes were used as background reference. Significance for each individual GO-identifier was computed with Fisher's exact test and a significance threshold of 1%. As GO terms are nonindependent, we used the ‘elim’ method in topGO, which integrates GO graph topography to improve enrichment analysis by iteratively removing genes mapped to significant GO terms from higher (more general) terms and reduces the rate of false positives (Alexa *et al*. 2006). Only GO terms having more than four and less than 1000 associated genes were considered in further analyses.

## Results

### Genome resequencing and Illumina read processing

Sequencing of five population pools resulted in 642 423 589 paired-end reads, corresponding to 128.5 Gb of sequence data. After adaptor and quality trimming, we retained 630 679 004 paired-end reads (98.2%) with a median Phred-score of 37 (mean = 125.8 million paired-end reads per pool; range: 103–141). This corresponds to an average 99x coverage per population pool.

### Read mapping and SNP calling

We could unambiguously map 18.3% of all *A. halleri* reads to the *A. thaliana* reference genome with low variation between the five population pools (range: 17.9–18.7%). One reason for this moderate proportion of mapped reads is that the *A. halleri* genome is more than twice the size of the *A. thaliana* reference genome. Consequently, we can expect to map less than 50% of our reads. Further, we discarded all reads mapping to organellar genomes (9.2–14.6% of the reads depending on the population), leaving approximately 35% of all reads. Because of the divergence between the two species, the remaining reads mapped primarily to conserved genic regions rather than to variable intergenic regions, resulting in ∼18% of mapped reads.

**Figure 2:**
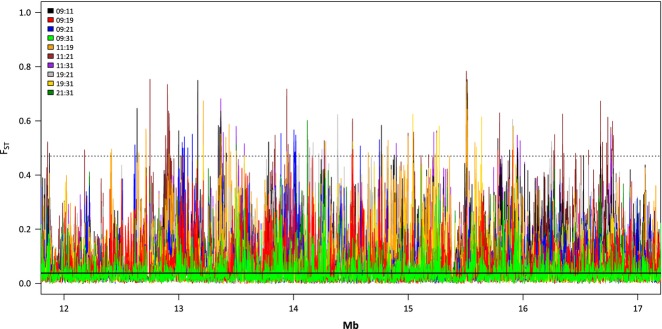
Results of the ten pairwise *F*_ST_ sliding-window analyses in *Arabidopsis halleri* along a 5 Mb stretch of *A. thaliana* chromosome 2. The solid line at 0.038 indicates the average differentiation across all SNPs, and the dotted line at 0.47 represents the 99.9% quantile threshold for strongly differentiated sliding windows.

Among the five populations studied, we identified 2 091 957 SNPs with a minimum allele count of four. Pairwise *F*_ST_ among populations based on all polymorphic SNPs was moderate, with an average of 0.038 and a range of 0.026 (Aha09 vs. 31) to 0.048 (Aha11 vs. 21; Table S2, Supporting information). The genomewide distribution of pairwise *F*_ST_ values (sliding-window values) is shown along a 5 Mb example section of chromosome 2 (Fig.2). The mean *F*_ST_ for sliding windows was slightly higher than for individual SNPs, with a mean of 0.057 and a range of 0.035 to 0.075. On average, we found 20.3 SNPs per sliding window, indicating that 4.06% of all covered nucleotide positions were variable.

Using the three-step procedure presented above, we identified 4282 strongly differentiated SNPs located within the 0.1% most strongly differentiated genomic regions. This corresponds to only 0.2% of all SNPs in the data set. Average sequence coverage for these SNPs was 60.3x (range: 51.5–70.3x). 204 SNPs could not be annotated and may be located in intergenic regions. The remaining SNPs resided in 571 unique genes (strongly differentiated SNPs per gene: average = 7.5, range = 1–146), 139 (24.3%) of which are assigned unknown function in TAIR10. The 0.1% most strongly differentiated sliding windows contained on average 12.96 SNPs. Polymorphism in these windows was thus significantly reduced compared with the average of 20.3 SNPs across all windows (Kruskal–Wallis test; *P *<* *2.2*10^−16^).

### Environmental association analyses

**Figure 3:**
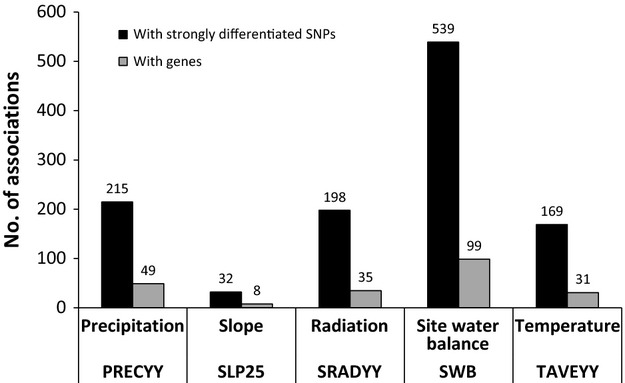
Numbers of associations for the 4282 most strongly differentiated SNPs (black columns) and genes (grey columns) with five environmental factors (Table S1, Supporting information) in *Arabidopsis halleri*.

Of the 21 410 partial Mantel tests performed between the 4282 highly differentiated SNPs and the five environmental factors, 1153 (5.4%) showed *r* values higher than the chosen threshold of 0.7573. Most associations were found with site water balance, followed by precipitation, radiation, temperature and slope (Fig.3). For 923 (89%) SNPs, an association with a single climatic factor was found, whereas 112 SNPs (10.8%) were associated with two, and two SNPs (0.2%) were associated with three environmental factors. In total, 175 (30.6%) of the 571 identified genes were strongly associated with at least one of the five topo-climatic factors studied (Table S3, Supporting information). Forty-one of these genes (23.4%) have unknown function according to TAIR10.

### Gene Ontology and functional annotation

By focusing on the GO categories that are most closely related to our five environmental factors, we arrived at an initial candidate gene list of 40 genes (‘Response to radiation’ = 19, ‘Response to osmotic stress’ = 9, ‘Response to temperature stimulus’ = 12 and ‘Response to water stimulus’ = 5, including five genes present in multiple categories). Of these, four genes had a clear association with environmental factors directly related to their putative biological function and were therefore considered as top candidate genes for adaptation to the abiotic environmental conditions studied in *A. halleri* (Table2).

**Table 2:** The four top candidate genes for associations with topo-climatic factors in *Arabidopsis halleri*. Given are the gene name, GO term, putative function, number of associated SNPs and number of nonsynonymous (NS) SNPs identified by the major allele consensus haplotype (MACH; see Materials and Methods). For abbreviations of topo-climatic factors, see Fig.3

Gene name	Response to	Putative function	SNP associations	NS
*LOS5/ABA3 (LOW OSMOTIC STRESS 5/ABA DEFICIENT 3; AT1G16540*)	Osmotic stress (GO:0006970)	Regulation of osmotic stress response (Xiong *et al*. 2002)	1xSWB	11
*PGP1/ABCB1* (*P-GLYCOPROTEIN 1/ATP-BINDING CASSETTE B1; AT2G36910*)	Radiation (GO:0009314)	Mediates hypocotyl length in response to light (Sidler *et al*. 1998)	52xSRADYY; 12xSWB	3
*GPX3* (*GLUTATHIONE PEROXIDASE 3*; *AT2G43350*)	Water stimulus (GO:0009415)	Scavenger in plant hormone ABA, H_2_O_2_ homeostasis and drought stress signalling (Miao *et al*. 2006)	10xSWB	3
*ATGLR 3.6* (*GLUTAMATE RECEPTOR 3.6*; *AT3G51480*)	Radiation (GO:0009314)	Potentially involved in light-signal transduction (Lacombe *et al*. 2001)	23xSRADYY; 2xSWB	11

Two of these genes in the GO category ‘response to radiation’ were associated with radiation (SRADYY). The first, *P-GLYCOPROTEIN 1 (PGP1)/ATP-BINDING CASSETTE B1 (ABCB1)* (AT2G36910), contained 64 strongly differentiated SNPs, and the second, *ATGLR3.6* (AT3G51480), contained 25 strongly differentiated SNPs (Table2). The other two top candidate genes showed significant associations with site water balance (SWB), which is directly linked to their biological processes ‘water stimulus’ and ‘osmotic stress’. The first, *GLUTATHIONE PEROXIDASE 3 (GPX3)* (AT2G43350), contained ten strongly differentiated SNPs that were all associated with SWB, and the second, *LOW OSMOTIC STRESS 5 (LOS5)/ABA DEFICIENT 3 (ABA3)* (AT1G16 540), contained a single strongly differentiated SNP associated with SWB (Table2).

### Nucleotide and amino acid variation in candidate genes

**Figure 4:**
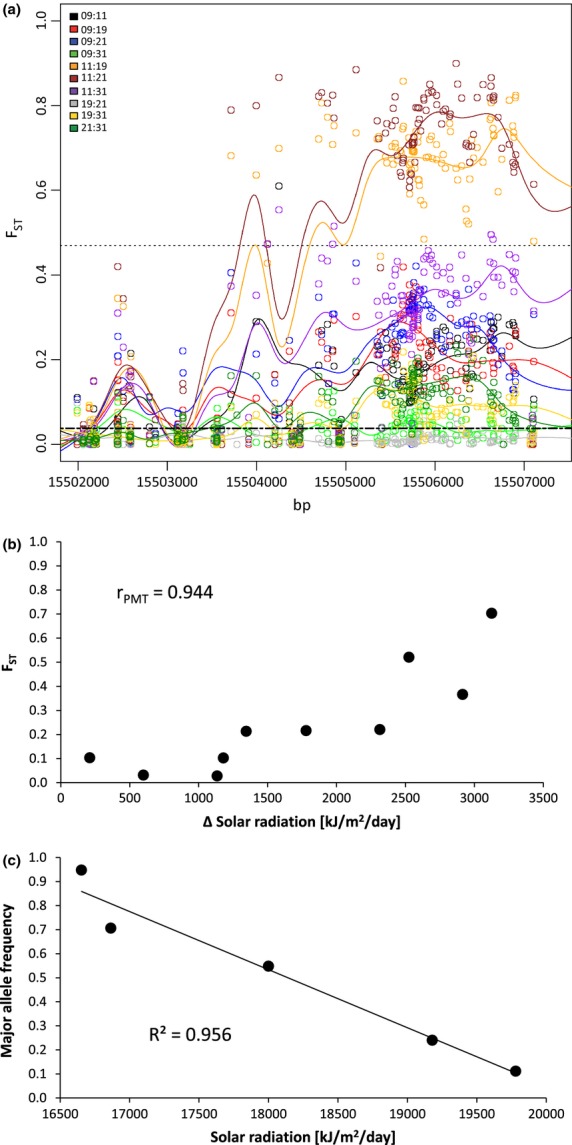
Population genomic evidence for adaptation to environmental variation in *A. halleri* for candidate gene *P-GLYCOPROTEIN 1* (AT2G36910). (a) Pairwise *F*_ST_ values from highly differentiated sliding windows (lines) and SNPs (open circles). The dashed line at 0.038 indicates the mean *F*_ST_ across all SNPs, and the dotted line at 0.47 represents the 99.9% quantile threshold for strongly differentiated sliding windows. (b) Correlation between pairwise population differences in *F*_ST_ and solar radiation. r_PMT_ represents the correlation coefficient of the partial Mantel test. (c) Linear regression between major allele frequencies and solar radiation. Results for the remaining candidate genes are shown in Fig. S1 (Supporting information).

Results of the *F*_ST_ and sliding-window analyses and environmental association plots are shown in Fig.4 for candidate gene *PGP1* and for the three other top candidate genes in Fig. S1 (Supporting information). The strongly differentiated regions span distances of at least 1000 bp (Fig.4a and Fig. S1a–c; Supporting information), which is consistent with a footprint of selection. Amino acid differences caused by nonsynonymous substitutions were found between MACHs (Table2). Interestingly, two of the nonsynonymous substitutions in genes *GPX3* and *ATGLR 3.6* were among the highly differentiated SNPs that are associated with the corresponding environmental factors.

### Gene Ontology enrichment analysis

Among the 571 genes containing strongly differentiated SNPs, we identified 16 significantly overrepresented biological processes (Table3). Responses to biotic factors, such as ‘defence response to bacterium’, played a prominent role. None of the GO categories directly related to the studied topo-climatic factors were found to be significantly overrepresented.

**Table 3:** The 16 top-ranked biological processes that were significantly overrepresented among the 571 genes containing highly differentiated SNPs in the five studied populations of *Arabidopsis halleri*

GO term	Biological process	Annotated^*^	Significant^†^	Expected^‡^	Fisher's test^§^
GO:0042742	Defence response to bacterium	211	13	4.95	0.0015
GO:0007568	Ageing	99	8	2.32	0.0023
GO:0007166	Cell surface receptor signalling pathway	149	10	3.49	0.0028
GO:0010214	Seed coat development	14	3	0.33	0.0038
GO:0046777	Protein autophosphorylation	86	7	2.02	0.0041
GO:0006820	Anion transport	86	7	2.02	0.0041
GO:0009863	Salicylic acid mediated signalling pathway	30	4	0.7	0.005
GO:0055085	Transmembrane transport	459	20	10.76	0.0062
GO:0051187	Cofactor catabolic process	32	4	0.75	0.0064
GO:0016568	Chromatin modification	95	7	2.23	0.007
GO:0048544	Recognition of pollen	33	4	0.77	0.0071
GO:0009740	Gibberellic acid mediated signalling pathway	35	4	0.82	0.0088
GO:0006306	DNA methylation	35	4	0.82	0.0088
GO:0006342	Chromatin silencing	19	3	0.45	0.0094
GO:2000280	Regulation of root development	19	3	0.45	0.0094
GO:0006487	Protein N-linked glycosylation	19	3	0.45	0.0094

* Number of genes within the background list which contain a specific GO term.

† Number of genes in the candidate list which have a specific GO term.

‡ Expected frequency (number of genes) of a given GO term based on the size of the candidate gene list.

§ *p*-value of the Fisher's exact test.

## Discussion

We studied genomewide polymorphisms in association with climatic variation in alpine populations of the emerging model plant *A. halleri*. Five geographically close populations from habitats characterized by diverse and distinct abiotic environmental conditions were investigated using a Pool-Seq genome resequencing approach. We observed more than 2 million SNPs among the populations and found a complex and highly diverse footprint of selection across the *A. halleri* genome. In a stepwise approach that included the identification of highly differentiated SNPs and genomic regions, environmental associations and GO term analyses, we identified a set of candidate genes associated with climatic variation. For four genes, genetic differentiation between populations and population allele frequencies, as well as functional annotations, was congruently associated with solar radiation and water availability. Our results are consistent with a scenario in which these genes are associated with adaptation to local abiotic environmental conditions in the investigated *A. halleri* populations and provide testable hypotheses for further experimental analyses of the ecological role of these genes.

### Genome resequencing and genomic signatures of selection

Pool-Seq genome resequencing used in this study was a cost-effective and powerful approach to assess genetic variation in natural populations and to identify genes that are potentially under selection, as previously suggested by both theoretical (Futschik & Schlötterer 2010; Gautier *et al*. 2013) and empirical studies (e.g. Fabian *et al*. 2012). The high coverage per population achieved in our study was adequate for reliable SNP calling and estimation of allele frequencies, as demonstrated in natural populations of *A. halleri* by Rellstab *et al*. (in press), where the average difference in population allele frequencies derived from pooled or individual samples was only 3.8% ± 0.8 SE.

Our analyses were strongly facilitated by the availability of the high-quality *A. thaliana* reference genome (Kaul *et al*. 2000), although the two species have different chromosome numbers and the *A. halleri* genome is approximately twice the size of the *A. thaliana* genome (Johnston *et al*. 2005). Nevertheless, sequence divergence in coding sequences between the two species was sufficiently low so that *A. halleri* reads could be mapped to the *A. thaliana* reference genome. A consequence of this mapping approach was that we assessed genetic variation mainly in relatively conserved coding regions, but it still allowed us to investigate genetic variation in >25 000 genes. Therefore, the results presented here are most likely not biased as a consequence of our mapping against the *A. thaliana* genome. From an evolutionary perspective, coding sequences are key genomic regions to look for signatures of selection, as these directly influence the protein function, in contrast to the often highly repetitive noncoding genome regions. Selection may also act on SNPs in noncoding regions as these may be located, for example, in promoters, enhancers or small RNAs where they affect gene expression. In this context, the excluded 204 SNPs that presumably reside in noncoding regions may be of interest for future studies.

We used a stepwise procedure to identify a handful of top candidate genes, starting with the identification of strongly differentiated SNPs and genomic regions (Kofler *et al*. 2011), taking into account population genetic structure. We assumed that highly differentiated regions of the genome are likely to be under selection, either directly or through hitchhiking (Strasburg *et al*. 2012), although we acknowledge that other processes, including reduced recombination, inversions and chromosomal rearrangements, may also lead to highly differentiated genomic regions or SNPs (Yeaman 2013). This approach substantially differs from the commonly used outlier detection approaches based on models of neutral evolution, which have been widely used to analyse predominantly anonymous markers (Excoffier *et al*. 2009; Fischer *et al*. 2011). However, our approach allowed us to take stochastic variation in sequence coverage at each SNP into account, a central aspect when using NGS data. In addition, we applied a stringent threshold for accepting highly differentiated SNPs (*F*_ST_ > 0.54). Consequently, *F*_ST_ for these SNPs was about 15 times higher than the average genomewide value (*F*_ST_ = 0.038). Moreover, SNPs of interest had to be located in highly differentiated genomic regions identified through sliding-window analysis. We thus focused on 4282 strongly differentiated SNPs that represented only 0.2% of all the SNPs detected and were located in 571 genes. The more than two million SNPs detected in this genome resequencing study therefore allowed us to be much more conservative than earlier genome scan studies based on AFLPs or SSRs, which often inferred 5–10% of markers to be under selection (Nosil *et al*. 2009).

The most strongly differentiated genomic regions in *A. halleri* were typically small (Fig.4) and widely dispersed across the genome (Fig.2), as observed in other organisms (Strasburg *et al*. 2012). This suggests that genomic divergence between locally adapted populations can be complex and is not limited to a few genomic areas. Further, many population pairs showed specific footprints of selection, suggesting that selection is often acting locally under heterogeneous environmental conditions and supporting the importance of standing genetic variation for adaption (Barrett & Schluter 2008). The complexity of adaptation to heterogeneous environments was corroborated by the GO enrichment analysis. It revealed a diverse range of overrepresented Gene Ontology categories (Table3), suggesting that both abiotic and biotic processes (e.g. plant–plant interactions or plant–pathogen interactions) contribute to adaptation to heterogeneous environments.

### Environmental associations

Our environmental association analyses revealed a strong association with at least one of the five investigated abiotic environmental factors for 30.6% of the genes showing footprints of selection. These associations suggest that precipitation (i.e. precipitation *per se* and site water balance), as well as solar radiation and temperature, affected adaptation in the *A. halleri* populations studied (Fig.3). The importance of these environmental factors in driving plant adaptation has also been inferred in other studies (e.g. Poncet *et al*. 2010; Fournier-Level *et al*. 2011; Hancock *et al*. 2011; Manel *et al*. 2012), but the identification of candidate genes underlying responses to these abiotic factors has been restricted to studies exploring sequence variation in candidate genes (e.g. Medina *et al*. 2011) or, on a genomewide scale, in the model plant *A. thaliana* (Fournier-Level *et al*. 2011; Hancock *et al*. 2011; Gaut 2012).

Alpine areas are predestined for studies of plant adaptation to environmental factors because abiotic and biotic conditions can substantially differ between populations situated in close proximity, while genetic effects due to demographic history are at a minimum. For example, our two populations from the surroundings of Vicosoprano were only 2 km apart, had low overall genetic differentiation (Table S2, Supporting information), but were located in habitats with pronounced environmental differences (Fig.1, Table1). Accordingly, we found several peaks of genetic differentiation between these two populations (Fig.2: blue line indicates differentiation between populations Aha09 and Aha21) even though gene flow may be ongoing over such small geographical scales. This supports our assumption that patterns of increased genetic differentiation in specific genomic areas mark the footprints of selection.

An important decision to take in environmental association analyses is what type of environmental data to use. The climatic factors used in this study represent long-term averages of environmental conditions collected along a dense network of meteorological stations over a period of 30 years (Zimmermann & Kienast 1999) and, like the topographical factors, are available at high resolution. We feel that such data are best suited for analyses of allele frequency changes leading to adaptation over multiple generations in perennial plants. In contrast, *in situ* measurements of abiotic conditions are informative on much smaller spatial or temporal scales and may be best suited for gene expression studies investigating immediate responses of plants to differences in environmental conditions.

### Candidate genes and their link to environmental factors

We found four top candidate genes that were strongly associated with abiotic environmental factors that are congruent with their assigned gene functions. Two of these candidate genes function in ‘response to radiation’, and our SNP allele frequencies showed a strong association with radiation (SRADYY). Gene *ATGLR3.6* is a member of a putative ligand-gated ion channel subunit family that may be involved in light-signal transduction and calcium homeostasis via the regulation of calcium influx into cells (Lacombe *et al*. 2001). The second gene, *PGP1/ABCB1,* encodes an auxin transporter (Petrasek *et al*. 2006) and appears to mediate hypocotyl length in response to light in *A. thaliana*. Transgenic plants overexpressing this gene develop longer hypocotyls (Sidler *et al*. 1998) and roots than wild-type individuals. This effect on root length may underlie the association with SWB.

*LOS5/ABA3,* associated with ‘osmotic stress’, is involved in the synthesis of the plant hormone ABA and appears to function in regulation of osmotic stress response (Xiong *et al*. 2002) and salt tolerance (Gao *et al*. 2011). *GPX3* is associated with ‘response to water stimulus’ functions in the ABA pathway and may have a dual function in *A. thaliana*, being involved in H_2_O_2_ homeostasis and relaying the H_2_O_2_ signal as an oxidative signal transducer in ABA and drought stress signalling. Deficiency and overexpression of *GPX3* in *A. thaliana* reduces and enhances drought stress tolerance, respectively (Miao *et al*. 2006).

These four top candidate genes are a small subsample of the 175 genes detected that contain highly differentiated SNPs and are strongly associated with at least one environmental factor studied (Table S3, Supporting information). However, the congruence between the observed environmental associations and the genes' assigned biological functions makes them ideal targets for further experimental validation, for example through transformation and genetic crosses with other *Arabidopsis* species (e.g. Hanikenne *et al*. 2008).

We hypothesize that genetic variation in the top candidate genes identified in this study has been shaped by natural selection. Alternatively, the identified genes could be hitchhiking genes (Strasburg *et al*. 2012), meaning that the targets of selection are not the identified genes themselves, but neighbouring genes or genetic elements. While this possibility cannot be excluded without further experimental data, several lines of evidence argue against this possibility. First, *A. halleri* is self-incompatible and thus an obligate outcrosser. In such species, linkage disequilibrium (LD), which provides the mechanistic basis for hitchhiking, is expected to be low. Our observation that patterns of elevated genetic differentiation often decay after a few thousand base pairs argues against hitchhiking (e.g. see Fig.4a). Second, our top candidate genes have functional annotations that are in line with the observed strong associations with climatic conditions. Such a pattern would not be expected for hitchhiking genes. Nevertheless, it will be of interest to assess the evidence for hitchhiking in detail once a well-annotated reference genome for *A. halleri* is available.

It is generally difficult to assess the potential role in adaptation of genes identified in population genomic analyses. To illustrate this point, more than 23% of the genes in our study containing highly differentiated SNPs have unknown functions, even in the most recent TAIR update (Lamesch *et al*. 2012). In addition, the functions of many other genes have not been studied in detail to date. Genome resequencing studies, such as the one presented here, can provide an unbiased perspective on potential targets of selection and may identify hitherto poorly studied genes with important ecological functions. The 175 genes carrying footprints of selection and having strong environmental associations (Table S3, Supporting information) found in this study therefore open the possibility for ecological gene annotation in natural populations and may motivate the validation and functional analysis of hitherto poorly studied genes that affect fitness in natural populations.

### Conclusions and outlook

The study of plant adaptation and genome evolution is an emerging field that is developing rapidly and offers countless opportunities to investigate adaptive processes. Here, we used a stepwise approach to identify genomic regions and candidate genes potentially involved in adaptation. The identified genes showed footprints of selection and were correlated with environmental factors that differed between sites, as expected under a scenario of environment-mediated selection in natural populations of *A. halleri*. The genomic distribution and assigned functions of the identified genes were as heterogeneous as the environmental conditions under which the sampled populations live. This may indicate that the evolution of geographically proximate populations that are potentially connected by gene flow, but are adapted to local environmental conditions, can have a complex genetic basis. Ideally, studies of adaptation should not solely rely on genetic variation, even on a genomewide scale, but should include experiments to study gene function at the molecular level and field transplant experiments to assess the adaptive value of candidate alleles and genes under realistic environmental conditions. This study helps formulating testable hypotheses for future experimental analyses of the ecological role of the identified genes in natural populations of alpine plants.

## Appendix

A.W., K.K.S. and R.H. conceived the project and obtained funding; all authors jointly designed the study; C.R., F.G., M.C.F. and A.W. conducted field-work; M.C.F., C.R., A.T. and S.Z. performed the research and analysed data; M.C.F., C.R., A.T. and A.W. wrote the manuscript with substantial contributions from S.Z., F.G., K.K.S. and R.H.

## Data accessibility

Filtered SNP loci files from PoPoolation2 and the perl script for the MACH analysis are available under DRYAD entry, doi: 10.5061/dryad.b2k47. RAW reads have been deposited in the Sequence Read Archive (SRP029378).

## Supporting Information

Fig S1 Results for the three candidate genes *LOS5/ABA3* (a,d,g), *GPX3* (b,e,f) and *ATGLR 3.6* (c,f,i). (a,b,c) represent pairwise *F*_ST_ values from highly differentiated sliding windows (lines) and SNPs (open circles).

Table S1 Description of the five topo-climatic factors used in the environmental association analyses of *Arabidopsis halleri*. **Table S2** Average genome-wide *F*_ST_ of pairwise population comparisons in *Arabidopsis halleri*, and threshold for accepting strongly differentiated SNPs. **Table S3** The 175 identified genes covering the highly differentiated SNPs that have at least one association with one or more of the five environmental factors studied (see Table S1, Supporting information).
