# Supplementary figures and images for: Population genomic footprints of selection and associations with climate in natural populations of Arabidopsis halleri from the Alps
Source: Mol Ecol. 2013 Oct 28;22(22):5594–607. doi: 10.1111/mec.12521 (PMC4274019; doi:10.1111/mec.12521)

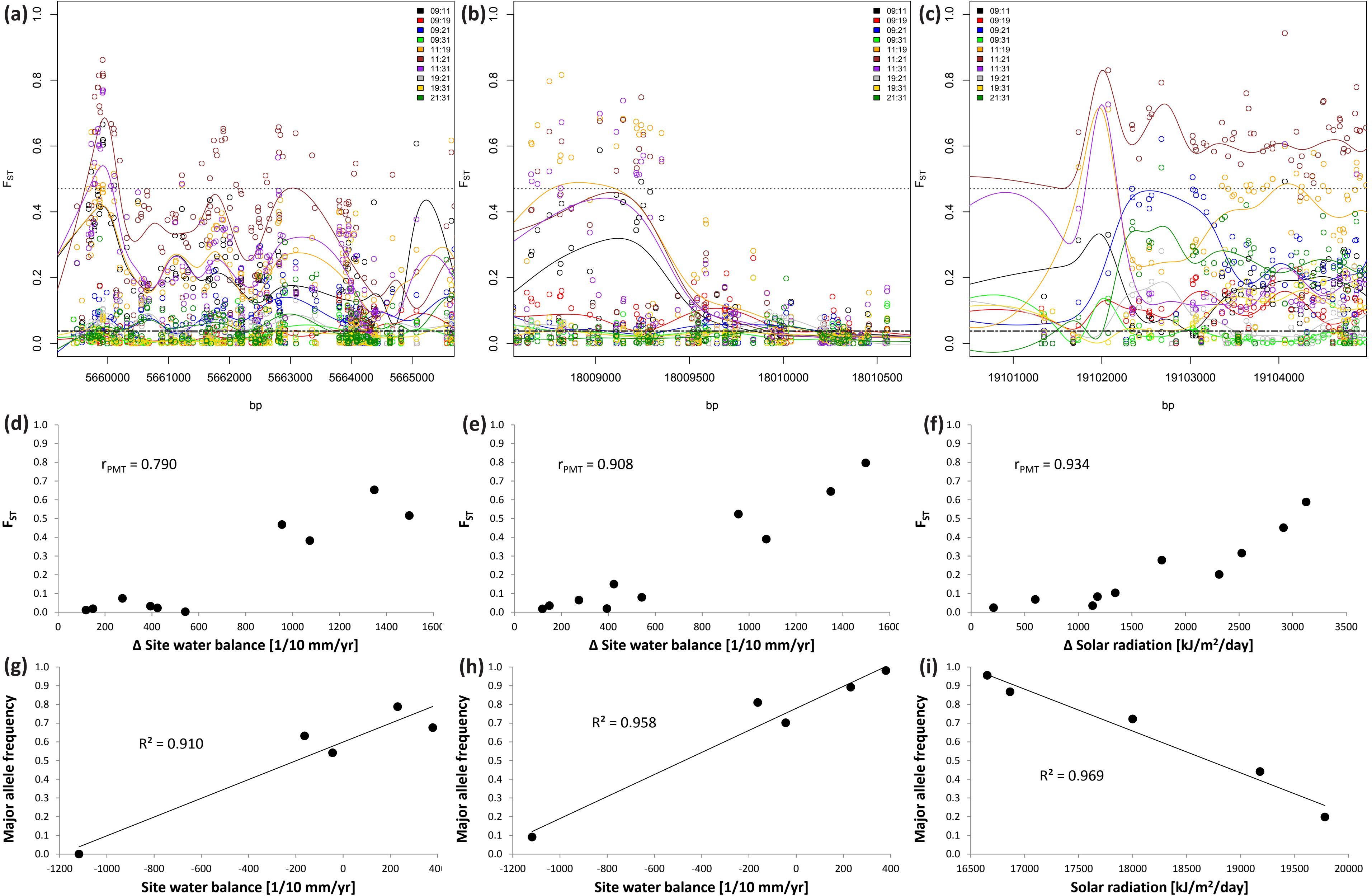

Supplement: Fig S1 — Results for the three candidate genes LOS5/ABA3 (a,d,g), GPX3 (b,e,f) and ATGLR 3.6 (c,f,i). (a,b,c) represent pairwise FST values from highly differentiated sliding windows (lines) and SNPs (open circles). [file mec0022-5594-sd1.pdf]
