# Supplementary material for: Population genomic footprints of selection and associations with climate in natural populations of Arabidopsis halleri from the Alps
Source: Mol Ecol. 2013 Oct 28;22(22):5594–607. doi: 10.1111/mec.12521 (PMC4274019; doi:10.1111/mec.12521)
Supplement: Table S1 — Description of the five topo-climatic factors used in the environmental association analyses of Arabidopsis halleri. Table S2 Average genome-wide FST of pairwise population comparisons in Arabidopsis halleri, and threshold for accepting strongly differentiated SNPs. Table S3 The 175 identified genes covering the highly differentiated SNPs that have at least one association with one or more of the five environmental factors studied (see Table S1, Supporting information). [file mec0022-5594-sd2.docx]

**Table S1** Description of the five topo-climatic factors used in the environmental association analyses of *Arabidopsis halleri*. All factors are available at a resolution of 25 m and were interpolated and averaged from meteorological measurements collected over a 30-year period between 1961 and 1990 ([Zimmermann & Kienast 1999](#_ENREF_2)).

| Environmental factor | Abbreviation | Description | Unit | In this study correlated with |
| --- | --- | --- | --- | --- |
| Precipitation | PRECYY | Yearly precipitation sum (rain and snow) | 1/10 mm/yr | Moisture index |
| Slope | SLP25 | Slope | ° | Aspect* |
| Solar radiation | SRADYY | Annual mean of daily global potential shortwave radiation | kJ/m^2^/day | Topographic position, topographic wetness index* |
| Site water balance | SWB | Annual average site water balance (= precipitation – evapotranspiration) | 1/10 mm/yr | Moisture index, topographic wetness index* |
| Temperature | TAVEYY | Mean average annual temperature | 1/100 °C | Altitude*, cloudiness, degree days, frost*, moisture index*, precipitation days, topographic position*, topographic wetness index |

* Negatively correlated factors.

**Table S2** Average genome-wide *F*_ST_ of pairwise population comparisons in *Arabidopsis halleri*, and threshold for accepting strongly differentiated SNPs. To take population structure into account, the thresholds have been adjusted by the pairwise neutral population genetic differentiation.

| Comparison | mean *F_ST_* | SNP *F*_ST_ threshold |
| --- | --- | --- |
| Aha 09:11 | 0.042 | 0.555 |
| Aha 09:19 | 0.034 | 0.547 |
| Aha 09:21 | 0.035 | 0.548 |
| Aha 09:31 | 0.026 | 0.539 |
| Aha 11:19 | 0.041 | 0.554 |
| Aha 11:21 | 0.048 | 0.561 |
| Aha 11:31 | 0.044 | 0.557 |
| Aha 19:21 | 0.037 | 0.550 |
| Aha 19:31 | 0.035 | 0.548 |
| Aha 21:31 | 0.036 | 0.549 |
| **Mean** | **0.038** | **0.545** |

**Table S3** The 175 identified genes covering the highly differentiated SNPs that have at least one association with one or more of the five environmental factors studied (see Table S1; Supporting information). Given are the gene IDs from TAIR10 ([Lamesch *et al.* 2012](#_ENREF_1)), the number of associations with environmental factors, and the putative gene function.

|  | **SNPs associated with** | | | | |  |
| --- | --- | --- | --- | --- | --- | --- |
|  | **Precipitation** | **Slope** | **Radiation** | **Site water balance** | **Temperature** | **Description** |
| **Gene ID** | (PRECYY) | (SLP25) | (SRADYY) | (SWB) | (TAVEYY) |  |
| **AT1G04510** |  |  |  | 2 |  | Pre-mRNA-processing factor 19 |
| **AT1G05540** | 1 |  |  |  |  | hypothetical protein |
| **AT1G05700** | 3 |  |  | 8 |  | Leucine-rich repeat transmembrane protein kinase protein |
| **AT1G06000** | 1 |  |  |  |  | UDP-glycosyltransferase-like protein |
| **AT1G06170** | 1 |  |  |  |  | transcription factor bHLH89 |
| **AT1G06960** |  |  |  |  | 1 | RNA recognition motif-containing protein |
| **AT1G06970** | 1 |  |  |  | 2 | cation/H(+) antiporter 14 |
| **AT1G09020** |  |  | 1 | 1 |  | sucrose nonfermenting 4-like protein |
| **AT1G09190** |  |  |  | 7 | 1 | pentatricopeptide repeat-containing protein |
| **AT1G09970** |  |  |  | 6 |  | leucine-rich receptor-like protein kinase |
| **AT1G10380** |  |  |  | 3 |  | Putative membrane lipoprotein |
| **AT1G11905** | 1 |  |  | 2 |  | B-cell receptor-associated protein 31-like protein |
| **AT1G15050** |  | 1 |  |  |  | auxin-responsive protein IAA34 |
| **AT1G16460** |  |  | 2 |  |  | rhodanese homologue 2 |
| **AT1G16540** |  |  |  | 1 |  | Molybdenum cofactor sulfurase |
| **AT1G20200** |  |  |  | 4 |  | 26S proteasome regulatory subunit N3 |
| **AT1G20500** |  |  | 5 |  |  | 4-coumarate--CoA ligase-like 4 |
| **AT1G24095** | 1 |  |  |  |  | Putative thiol-disulfide oxidoreductase DCC |
| **AT1G30290** |  |  | 1 |  |  | PPR repeat domain-containing protein |
| **AT1G30330** |  |  | 10 |  |  | auxin response factor 6 |
| **AT1G48090** | 1 |  |  |  |  | calcium-dependent lipid-binding-like protein |
| **AT1G52900** |  |  |  | 2 |  | Toll-Interleukin-Resistance domain-containing protein |
| **AT1G53050** |  |  | 2 | 1 |  | protein kinase-like protein |
| **AT1G53460** | 4 |  |  |  | 1 | hypothetical protein |
| **AT1G53470** | 2 |  |  |  |  | mechanosensitive channel of small conductance-like 4 |
| **AT1G53500** | 1 |  |  |  |  | UDP-glucose 4,6-dehydratase |
| **AT1G53520** | 14 |  |  | 1 |  | chalcone isomerase-like protein |
| **AT1G60860** |  |  |  | 5 |  | ADP-ribosylation factor GTPase-activating protein AGD2 |
| **AT1G61240** | 3 |  | 2 |  | 5 | lysine ketoglutarate reductase trans-splicing related 1 |
| **AT1G64010** |  |  |  |  | 10 | serine protease inhibitor-like protein |
| **AT1G64070** |  |  |  |  | 1 | TIR-NBS-LRR class disease resistance protein |
| **AT1G64610** |  |  |  | 1 |  | WD40 domain-containing protein |
| **AT1G65730** |  |  | 1 |  |  | putative metal-nicotianamine transporter YSL7 |
| **AT1G68720** |  |  | 4 | 2 | 15 | tRNA-specific adenosine deaminase |
| **AT1G69720** |  |  |  | 1 |  | heme oxygenase 3 |
| **AT1G78650** |  |  |  | 1 |  | DNA-directed DNA polymerase |
| **AT1G80560** |  |  | 1 |  |  | 3-isopropylmalate dehydrogenase 2 |
| **AT1G80580** |  |  | 1 |  |  | ethylene-responsive transcription factor ERF084 |
| **AT1G80660** |  |  |  | 1 |  | H(+)-ATPase 9 |
| **AT1G80680** |  |  | 1 | 2 |  | suppressor of auxin resistance 3 |
| **AT1G80690** |  |  |  | 1 |  | PPPDE putative thiol peptidase family protein |
| **AT1G80740** |  |  |  | 1 |  | putative DNA (cytosine-5)-methyltransferase CMT1 |
| **AT1G80750** |  |  |  | 13 |  | 60S ribosomal protein L7-1 |
| **AT2G17050** |  |  |  | 1 |  | TIR-NBS-LRR class disease resistance protein |
| **AT2G19600** |  |  |  | 1 |  | K(+) efflux antiporter 4 |
| **AT2G19880** | 1 |  |  | 3 |  | nucleotide-diphospho-sugar transferase domain-containing protein |
| **AT2G20960** |  |  |  | 3 |  | phospholipase-like protein (PEARLI 4) domain-containing protein |
| **AT2G20970** |  |  |  | 3 |  | putative lipid binding protein |
| **AT2G21500** |  |  |  | 5 |  | RING/U-box domain-containing protein |
| **AT2G21680** |  |  | 2 |  |  | Kelch motif-containing protein |
| **AT2G21770** |  |  | 2 |  |  | cellulose synthase A |
| **AT2G21900** |  |  |  | 6 |  | putative WRKY transcription factor 59 |
| **AT2G21950** |  |  |  | 2 |  | F-box/kelch-repeat protein SKIP6 |
| **AT2G21960** |  |  |  | 1 |  | hypothetical protein |
| **AT2G22070** |  |  |  | 3 |  | pentatricopeptide repeat-containing protein |
| **AT2G22090** |  |  |  | 3 |  | UBP1 interacting protein 1a |
| **AT2G22680** |  |  | 2 | 31 |  | C3HC4-type RING finger domain-containing protein |
| **AT2G23350** |  |  |  |  | 1 | poly(A) binding protein 4 |
| **AT2G23390** |  |  |  | 1 |  | hypothetical protein |
| **AT2G23460** |  |  |  | 3 |  | extra-large G-protein 1 |
| **AT2G23790** |  |  |  | 3 |  | hypothetical protein |
| **AT2G23810** |  |  |  | 5 |  | tetraspanin8 |
| **AT2G24617** |  |  |  | 1 |  | hypothetical protein |
| **AT2G25410** |  |  |  | 1 |  | RING-H2 finger protein ATL22 |
| **AT2G28490** |  |  | 3 |  |  | cupin domain-containing protein |
| **AT2G28890** |  |  |  | 2 |  | putative protein phosphatase 2C 23 |
| **AT2G29510** |  |  |  | 2 |  | hypothetical protein |
| **AT2G29760** | 1 |  |  |  |  | pentatricopeptide repeat-containing protein |
| **AT2G30280** |  |  | 14 | 2 |  | RNA-directed DNA methylation 4 |
| **AT2G30290** |  |  | 3 | 1 |  | vacuolar-sorting receptor 2 |
| **AT2G30300** |  |  | 4 | 1 |  | major facilitator protein |
| **AT2G30590** |  | 2 |  |  |  | WRKY DNA-binding protein 21 |
| **AT2G30780** |  |  | 1 |  |  | pentatricopeptide repeat-containing protein |
| **AT2G31060** | 8 |  |  |  | 8 | elongation factor-like protein |
| **AT2G31570** | 11 |  |  |  |  | glutathione peroxidase |
| **AT2G31620** | 2 |  |  |  |  | putative cysteine-rich repeat secretory protein 10 |
| **AT2G32860** |  |  | 1 |  |  | beta glucosidase 33 |
| **AT2G33010** |  | 2 |  |  |  | Ubiquitin-associated (UBA) protein |
| **AT2G33040** |  | 6 |  |  |  | ATP synthase subunit gamma |
| **AT2G33150** |  | 1 |  |  |  | 3-ketoacyl-CoA thiolase 2 |
| **AT2G33670** |  | 2 |  |  |  | MLO-like protein 5 |
| **AT2G35140** |  | 6 |  |  |  | DCD (Development and Cell Death) domain protein |
| **AT2G35160** |  |  |  | 1 |  | histone-lysine N-methyltransferase, H3 lysine-9 specific SUVH5 |
| **AT2G35800** |  |  |  | 11 |  | mitochondrial substrate carrier family protein |
| **AT2G36190** |  |  |  | 2 |  | beta-fructofuranosidase |
| **AT2G36330** |  |  |  |  | 1 | hypothetical protein |
| **AT2G36370** |  |  |  | 1 | 6 | ubiquitin-protein ligase |
| **AT2G36910** |  |  | 52 | 12 |  | ABC transporter B family member 1 |
| **AT2G36950** |  |  |  | 2 |  | heavy-metal-associated domain-containing protein |
| **AT2G37070** |  |  |  | 9 | 12 | hypothetical protein |
| **AT2G37110** |  |  |  |  | 1 | PLAC8 domain-containing protein |
| **AT2G38010** | 7 |  |  |  |  | neutral ceramidase |
| **AT2G41460** | 16 |  |  | 7 | 8 | apurinic endonuclease-redox protein |
| **AT2G41890** | 3 |  |  | 1 |  | curculin-like mannose-binding lectin fand PAN domain-containing protein |
| **AT2G42330** |  |  |  | 31 |  | GC-rich sequence DNA-binding factor-like protein with Tuftelin interacting domain |
| **AT2G42440** |  |  |  | 5 |  | LOB domain-containing protein 17 |
| **AT2G42450** |  |  |  | 12 |  | alpha/beta-hydrolase domain-containing protein |
| **AT2G42560** |  |  |  | 3 |  | late embryogenesis abundant domain-containing protein |
| **AT2G42570** |  |  |  | 10 |  | protein trichome birefringence-like 39 |
| **AT2G42600** | 1 |  |  | 2 |  | phosphoenolpyruvate carboxylase 2 |
| **AT2G43255** | 4 |  |  |  |  | hypothetical protein |
| **AT2G43350** |  |  |  | 10 |  | putative glutathione peroxidase 3 |
| **AT2G43370** |  |  |  | 18 |  | U11/U12 small nuclear ribonucleoprotein 35 kDa protein |
| **AT2G47010** |  |  |  |  | 2 | hypothetical protein |
| **AT3G19040** | 18 |  |  | 37 | 21 | transcription initiation factor TFIID subunit 1-B |
| **AT3G19050** | 20 |  |  | 57 | 36 | phragmoplast orienting kinesin 2 |
| **AT3G19220** | 1 |  |  | 1 |  | protein disulfide isomerase |
| **AT3G19230** | 1 |  |  |  |  | leucine-rich repeat-containing protein |
| **AT3G21480** |  |  | 9 |  |  | BRCT domain-containing DNA repair protein |
| **AT3G22425** | 2 |  |  |  | 1 | Imidazoleglycerol-phosphate dehydratase 1 |
| **AT3G22430** | 2 |  |  |  |  | hypothetical protein |
| **AT3G24160** | 4 |  |  |  |  | putative type 1 membrane protein |
| **AT3G26100** |  |  | 5 |  |  | regulator of chromosome condensation repeat-containing protein |
| **AT3G26115** |  |  | 12 |  |  | Pyridoxal-5'-phosphate-dependent enzyme family protein |
| **AT3G26120** |  |  | 6 |  |  | terminal EAR1-like 1 |
| **AT3G26590** |  |  |  | 1 | 1 | mate efflux domain-containing protein |
| **AT3G26890** |  |  |  | 2 | 1 | hypothetical protein |
| **AT3G27020** |  |  |  | 7 | 5 | putative metal-nicotianamine transporter YSL6 |
| **AT3G28315** |  |  |  | 2 |  | pseudo |
| **AT3G28960** | 2 |  |  |  |  | Transmembrane amino acid transporter family protein |
| **AT3G48010** |  | 12 |  |  |  | cyclic nucleotide gated channel |
| **AT3G49250** | 2 |  |  |  |  | protein defective in meristem silencing 3 |
| **AT3G51390** |  |  | 1 |  |  | putative S-acyltransferase |
| **AT3G51410** |  |  | 10 |  |  | hypothetical protein |
| **AT3G51470** |  |  | 1 |  |  | putative protein phosphatase 2C 47 |
| **AT3G51480** |  |  | 23 | 2 |  | glutamate receptor 3.6 |
| **AT3G51490** |  |  | 4 |  |  | monosaccharide-sensing protein 3 |
| **AT3G51570** |  |  |  | 11 |  | TIR-NBS-LRR class disease resistance protein |
| **AT3G51740** |  |  |  | 2 |  | probably inactive leucine-rich repeat receptor-like protein kinase IMK2 |
| **AT3G51770** |  |  | 1 | 6 |  | tetratricopeptide repeat (TPR)-containing protein |
| **AT3G51850** |  |  |  | 1 |  | calcium-dependent protein kinase 13 |
| **AT3G51860** |  |  | 3 | 3 |  | vacuolar cation/proton exchanger 3 |
| **AT3G56430** |  |  | 1 |  |  | hypothetical protein |
| **AT3G56590** |  |  |  |  | 1 | hydroxyproline-rich glycoprotein family protein |
| **AT3G61750** |  |  |  | 1 |  | Cytochrome b561/ferric reductase transmembrane with DOMON related domain |
| **AT3G61760** |  |  |  | 27 |  | dynamin-related protein 1B |
| **AT4G00550** |  |  |  | 1 |  | digalactosyldiacylglycerol synthase 2 |
| **AT4G00570** |  |  | 1 |  |  | malate dehydrogenase (decarboxylating) |
| **AT4G02010** | 1 |  |  |  |  | protein kinase family protein |
| **AT4G09040** | 5 |  |  |  |  | RNA recognition motif-containing protein |
| **AT4G09160** |  |  |  | 1 |  | patellin-5 |
| **AT4G10120** |  |  |  | 1 |  | sucrose-phosphate synthase |
| **AT4G14220** |  |  |  | 9 |  | E3 ubiquitin-protein ligase RHF1A |
| **AT4G17650** | 3 |  |  |  |  | Polyketide cyclase / dehydrase and lipid transport protein |
| **AT4G20230** | 1 |  |  |  | 1 | terpene cyclase, C1 domain-containing protein |
| **AT4G21070** | 7 |  |  | 11 | 4 | protein BREAST CANCER SUSCEPTIBILITY 1-like protein |
| **AT4G21450** |  |  |  | 5 |  | vesicle-associated membrane family protein |
| **AT4G31520** | 4 |  |  |  |  | SDA1 family protein |
| **AT5G03570** |  |  |  | 4 |  | protein iron regulated 2 |
| **AT5G07130** |  |  |  | 2 |  | laccase 13 |
| **AT5G11720** |  |  |  | 1 |  | alpha-glucosidase |
| **AT5G12210** | 1 |  |  | 1 |  | RAB geranylgeranyl transferase beta subunit 1 |
| **AT5G13160** |  |  |  | 2 |  | serine/threonine-protein kinase PBS1 |
| **AT5G14570** |  |  |  | 1 |  | high affinity nitrate transporter 2.7 |
| **AT5G14950** | 1 |  |  |  |  | alpha-mannosidase II |
| **AT5G15340** |  |  |  | 2 |  | pentatricopeptide repeat-containing protein |
| **AT5G15440** | 4 |  |  | 1 |  | EID1-like F-box protein 1 |
| **AT5G15450** | 1 |  |  | 1 |  | casein lytic proteinase B3 |
| **AT5G37530** |  |  |  | 10 |  | Rossmann-fold NAD(P)-binding domain-containing protein |
| **AT5G42800** | 2 |  |  |  |  | dihydroflavonol-4-reductase |
| **AT5G43420** | 7 |  |  |  | 4 | RING-H2 finger protein ATL16 |
| **AT5G43430** | 7 |  |  |  | 1 | Electron transfer flavoprotein subunit beta |
| **AT5G43650** |  |  |  | 1 |  | transcription factor bHLH92 |
| **AT5G46270** |  |  |  | 1 |  | Disease resistance protein (TIR-NBS-LRR class) family protein |
| **AT5G47210** |  |  |  | 1 |  | hyaluronan / mRNA binding-like protein |
| **AT5G48170** |  |  |  | 1 |  | F-box protein SNE |
| **AT5G48360** |  |  |  | 6 |  | formin-like protein 9 |
| **AT5G56680** | 10 |  |  |  |  | asparaginyl-tRNA synthetase, cytoplasmic 1 |
| **AT5G56790** | 9 |  |  |  |  | protein kinase family protein |
| **AT5G56810** | 4 |  |  |  |  | putative F-box/FBD/LRR-repeat protein |
| **AT5G62230** |  |  |  |  | 1 | LRR receptor-like serine/threonine-protein kinase ERL1 |
| **AT5G63000** |  |  |  |  | 6 | Mitochondrial import inner membrane translocase subunit Tim17/Tim22/Tim23 family protein |
| **AT5G63140** |  |  |  |  | 7 | purple acid phosphatase 29 |
| **AT5G67230** | 1 |  |  |  | 1 | glycosyl transferase family 43 protein |
| **AT5G67500** |  |  |  | 3 |  | voltage dependent anion channel 2 |
| **No annotation (various genes)** | 7 |  | 6 | 26 | 3 |  |

**Figure S1:** Results for the three candidate genes *LOS5/ABA3* (a,d,g), *GPX3* (b,e,f) and *ATGLR 3.6* (c,f,i). (a,b,c) represent pairwise *F*_ST_ values from highly differentiated sliding windows (lines) and SNPs (open circles). The bold dotted line at 0.038 indicates the mean *F*_ST_ across all SNPs and the dotted line at 0.47 represents the 99.9% quantile threshold for strongly differentiated sliding windows. (d,e,f) show correlations between pairwise population differences in *F*_ST,_ and site water balance (d, e) or solar radiation (f). r_PMT_ represents the correlation coefficient of the partial Mantel test. (g,h,i) display linear regressions between major allele frequencies and site water balance (g, h) or solar radiation (i).

**References**

Lamesch P, Berardini TZ, Li D*, et al.* (2012) The *Arabidopsis* information resource (TAIR): improved gene annotation and new tools. *Nucleic Acids Research* **40**, D1202-D1210.

Zimmermann NE, Kienast F (1999) Predictive mapping of alpine grasslands in Switzerland: species versus community approach. *Journal of Vegetation Science* **10**, 469-482.
